# A Gamified Cardiometabolic Health Curriculum Utilizing a Student Response System for Internal Medicine Residents

**DOI:** 10.7759/cureus.87718

**Published:** 2025-07-11

**Authors:** Ji-Cheng (Jason) Hsieh, Zohaib Bagha, Spencer Weintraub, Jack Jnani, Lauren Block

**Affiliations:** 1 Internal Medicine, Northwell Health, New Hyde Park, USA; 2 Northwell Cardiovascular Institute, Northwell Health, New Hyde Park, USA

**Keywords:** cardiometabolic health, curriculum development and evaluation, gamification technique, medical education technology, medical resident education, resident doctor, virtual learning

## Abstract

Introduction: Cardiovascular disease is frequently addressed in primary care, and professional association guidelines dictating the standard of care frequently change. Teaching clinical practice guidelines to residents remains important to promote knowledge retention and subsequent implementation in patient care. Gamification utilizes social elements and technology to promote accessible and active learning that enhances learner confidence, engagement, and knowledge. Data assessing gamification in teaching guideline-based care remains limited. We evaluated a gamified cardiometabolic health curriculum utilizing a student response system (SRS) to assess the benefits of gamification in teaching guidelines to internal medicine residents.

Methods: We developed a gamified cardiometabolic health curriculum for categorical residents in internal medicine utilizing KAHOOT!® (Oslo, Norway). KAHOOT!® is an SRS where learners utilize their mobile devices to answer questions. Speed and accuracy contributed to placement on a digital leaderboard. Our curriculum consisted of one academic half-day with five 45-minute virtual didactics reviewing guideline-based management of type 2 diabetes mellitus (T2DM), hypertension, hyperlipidemia, metabolic-associated fatty liver dysfunction (MAFLD), and weight management. KAHOOT!® was selected to promote ease of participation. The gamified format was compared to a traditional slide-based format. Residents are randomly assigned one of five clinic groups. Three groups received the gamified format, and two received the traditional format, without crossover. Residents were invited to complete anonymous pre- and post-surveys, which collected quantitative data such as Likert scale ratings of confidence and satisfaction with learning and test performance.

Results: All residents (108 of 108) received the curriculum. Around 59% (64) received the gamified format, and 44 (41%) received the traditional format. Pre-post surveys were compared via a matched analysis. Anonymous surveys were matched via a code provided by each resident, and residents who completed only one of the two surveys were excluded. Likert scale ratings of confidence in management increased for hypertension, hyperlipidemia, MAFLD, and weight management, but not for T2DM, likely as T2DM had the lowest number of matched resident pairs analyzed. Comparing gamified and traditional formats, there was no difference in the post-curricular change in absolute score on board-style questions or increase in Likert scale ratings of confidence. A majority of residents (90.9%) who received the gamified format found it engaging, while a minority (14.2%) of residents who received the traditional format found it engaging. Analysis was limited by a low response rate of 21.6%. Anonymous pre-post surveys limited the assessment of non-response bias and whether respondent characteristics differed from those of non-respondents.

Discussion: A gamified format was similar to a traditional format in increasing resident confidence in managing cardiometabolic health conditions and knowledge of guidelines. A gamified format was felt to be more engaging. Although the low response rate limits generalizability, we present initial data supporting the efficacy of a virtual gamified curriculum utilizing an SRS to teach clinical practice guidelines. Future avenues for study include assessments of long-term knowledge retention and whether engagement translates to increased learner participation.

## Introduction

This article was previously presented as a meeting abstract at the 2024 American College of Cardiology Annual Scientific Session on April 7th, 2024. Cardiovascular disease prevention and management are commonly addressed in primary care [1]. Given that professional association guidelines dictating standards of care in promoting cardiovascular health frequently change, developing and evaluating novel educational modalities, such as gamification, remain important to developing effective educational curricula for residents. Gamification broadly encompasses a variety of systems, including social elements (team competition), technology integration (novel devices, virtual reality), and systems (point-based scoring or a fictional world) that promote engagement and accessible and active learning [2,3]. Social learning theory emphasizes that observation and imitation serve as a foundation of learning, and the framework of peer-to-peer competition established by gamification promotes this imitation among peers [4]. Student response systems (SRS) are a type of gamification described as a quiz-based system utilizing mobile devices, where speed and accuracy contribute to placement on a digital leaderboard [5].

Most gamification studies pertain to procedural skills, specifically in laparoscopic or robotic procedures, with an emphasis on competition in surgical specialties [6], but some report benefits in acquiring general medical knowledge [7,8]. Most prior studies utilize gamification systems such as role-playing [9,10], card games [11], and board games [8] to teach medical knowledge or specific skills such as task prioritization or billing. There remain limited studies assessing the role of gamification, specifically an SRS, in a live, interactive format [12]. There also remains no standardized curriculum for teaching clinical practice guideline-based care, and there remains limited data comparing gamification to traditional didactic lecture in medical knowledge of cardiometabolic health guidelines among residents [12]. Additionally, there remains a need for an educational format that can adapt to continual updates in practice guidelines. Our study aims to compare a gamified format utilizing KAHOOT!® (Oslo, Norway) with traditional slide-based didactic lecture in teaching evidence-based guidelines in cardiometabolic health to categorical internal medicine residents. We assessed a variety of learning outcomes, including post-curricular short-term knowledge retention as well as self-reported confidence and engagement with didactic content.

## Materials and methods

Curriculum description

The study was conducted at the Zucker School of Medicine at Hofstra/Northwell Program. Our cardiometabolic health curriculum was launched during the 2023-2024 academic year at a single large internal medicine residency program. The curriculum comprised five 45-minute didactic lectures delivered virtually over Zoom (Zoom Communications, Inc., San Jose, CA, USA). Each didactic utilized case-based review to summarize professional association guidelines (Table 1) pertaining to one topic in cardiometabolic health: type 2 diabetes mellitus (T2DM), hypertension, hyperlipidemia, metabolic-associated fatty liver dysfunction (MAFLD), and weight management. These five topics were selected to best represent cardiometabolic health conditions encountered in the ambulatory setting. Content pertaining to initial diagnostic testing, laboratory monitoring, and medication management was prioritized for inclusion in our curriculum, with particular focus given to new updates. A virtual format was selected due to institutional preference, as we aimed to integrate our cardiometabolic curriculum into a pre-existing virtual ambulatory curriculum that was delivered throughout the year during dedicated academic half-days.

**Table 1:** Guidelines included in the cardiometabolic health curriculum AACE: American Association of Clinical Endocrinology; AHA: American Heart Association; ACC: American College of Cardiology; ACE: American College of Endocrinology; AASLD: American Association for the Study of Liver Diseases

Topic Area	Guidelines Referenced in Our Cardiometabolic Health Curriculum
Type 2 Diabetes	AACE Consensus Statement: Comprehensive Type 2 Diabetes Management Algorithm and Pharmacologic Approaches to Glycemic Treatment: Standards of Care in Diabetes - 2023 [7,8].
Hyperlipidemia	2018 AHA/ACC Guideline on the Management of Blood Cholesterol, 2022 ACC Expert Consensus Decision Pathway on the Role of Nonstatin Therapies or LDL-Cholesterol Lowering in the Management of Atherosclerotic Cardiovascular Disease, and Statin Use for Primary Prevention of Cardiovascular Disease in Adults: US Preventative Services Task Force Recommendation Statement [9-11].
Hypertension	2017 ACC/AHA Guideline for the Prevention, Detection, Evaluation, and Management of High Blood Pressure in Adults [12].
Weight Management	AACE/ACE Comprehensive Clinical Practice Guidelines for Medical Care of Patients with Obesity [13].
Metabolic-Associated Fatty Liver Dysfunction	AASLD Practice Guidance on the Clinical Assessment and Management of Nonalcoholic Fatty Liver Disease [14].

Each didactic was developed by faculty, a chief resident, a senior resident volunteer, or a pharmacist and reviewed by ambulatory faculty. Sessions were primarily resident-led, with ambulatory faculty present to guide discussion and offer additional insights. All five didactics were mandatory as a part of a dedicated academic half-day during the ambulatory clinic week. Our residency program utilized a “4+1” “X+Y” system, where residents are organized into distinct clinic groups and alternate between four weeks of inpatient service and one week of ambulatory clinic. As a result, didactics were delivered in series to categorical PGY-1, PGY-2, and PGY-3 internal medicine residents in clinic groups of 20-25 residents.

Traditional didactics consisted of slide-based electronic sessions with Microsoft PowerPoint (Microsoft Corporation, Redmond, Washington, United States). Didactics were gamified using KAHOOT!®, an SRS. Residents answered questions individually, with their speed and accuracy contributing to their ranking on a leaderboard displayed after each question. Residents were motivated by the score itself; the highest-scoring resident did not receive any additional reward. KAHOOT!® was chosen as it has been shown to improve knowledge retention, skills learning, confidence, and motivation in primary and secondary education, as well as medical education [5,7]. Prior gamification studies of residents utilized card-based games with specific instructions and materials, and we sought to develop a streamlined gamified experience for the virtual setting with minimal instruction necessary [11]. Residents were able to utilize their personal cell phones without any additional equipment required, leveraging the prevalence of and resident familiarity with mobile devices to our benefit.

Participant recruitment

Inclusion criteria required participants to be PGY-1, PGY-2, or PGY-3 categorical medicine residents. Preliminary internal medicine residents, medical students, and faculty were excluded from the study. All residents who received the curriculum were invited to complete surveys via e-mail. Survey participation was strictly voluntary. Residents’ decision to participate had no bearing on performance evaluations or employment status. Placement on the leaderboard during the curriculum served as a social desirability incentive to participate in the curriculum. Otherwise, there were no specific incentives used to increase survey participation. However, participation was heavily encouraged by presenters and faculty, and residents attended the curriculum during protected academic time. An appropriate time was allocated during each session to complete surveys. Survey QR codes were embedded within presentations for ease of access. Additionally, weekly reminder e-mails also served to increase participation.

Data collection

At the start of residency in the program, PGY1 residents are assigned one of five clinic groups by random selection, and they remain in these clinic groups throughout their PGY2 and PGY3 years. The study leveraged this organizational system to assign three clinic groups to the gamified format and two groups to the traditional format without crossover. Residents received a pre-test, post-test, and end-of-year engagement survey. Surveys were designed to include primarily quantitative measures (Appendix 1). Pre- and post-test surveys included a set of five internal medicine board-style questions as a quantitative measure of knowledge and five-point Likert scales as a quantitative measure of self-reported knowledge of guidelines and confidence in managing each cardiometabolic health condition. One represented the least confident or knowledgeable, and five represented the most. There was no preparatory time given to residents before completing surveys in order to best assess the effect of our educational intervention on learning outcomes. Board-style questions included case vignettes that were not validated but drawn from question banks commonly used for the preparation of the American Board of Internal Medicine. The engagement survey included five-point Likert scales assessing both engagement and motivation to utilize guideline recommendations in clinical practice, ranging from “strongly disagree” to “strongly agree.” The engagement survey also included a question regarding preference for a gamified or traditional format for subsequent academic years. All residents within the program are required to complete monthly internal medicine board test preparation questions; the average proportion correct of these assigned questions was collected to measure the baseline academic performance of both gamified and traditional groups.

Data analysis

We utilized a matched analysis comparing pre- and post-surveys, with residents serving as their own control. To account for smaller sample sizes, we utilized a non-parametric approach that does not assume normality, with a dependent or paired analysis using the Wilcoxon matched-pairs signed rank test to assess for post-pre differences within matched groups. A matched analysis was also chosen to utilize the Mann-Whitney U test to compare differences in rank sums for two independent groups, such as post-pre differences in the gamified vs. traditional cohorts, which necessitated residents serving as their own control in order to determine post-pre differences in test performance and Likert scale ratings. Test performance is listed as a percentage of correct responses on a five-question test, and Likert scale ratings are listed as a number on a one-to-five scale. Two-tailed p-values were calculated; p-values <0.05 were statistically significant. Analysis was performed using GraphPad Prism (San Diego, CA). Informed consent was not collected from participants, as survey data remained anonymous and participation was optional. This study was exempt by the Northwell Health Institutional Review Board (Study Number 24-0406).

## Results

All 108 residents in the program met the inclusion criteria and participated. The gamified cohort contained 64 residents, and the traditional cohort contained 44 residents. Overall response rate across all five didactics was 21.6%, with a range from 12.9 to 37.9% depending on the didactic. Table 2 describes baseline demographics. Demographic measures of the gamified and traditional groups were generally similar at baseline.

**Table 2:** Study population demographics

Characteristic	Gamified	Non-gamified
Total number of residents	64	44
Average resident age (Years)	29.3	29.9
Proportion of PGY-1 residents (%)	32.8%	34.1%
Proportion of female residents (%)	35.9%	29.6%
Average proportion correct of previously assigned internal medicine board test preparation questions (%)	55.5%	55.4%

Type 2 diabetes mellitus didactic

Around 14/108 (12.9%) total matched pairs in the gamified and traditional cohorts completed all pre- and post-surveys. Combined data including both cohorts demonstrated a non-significant increase in median test performance on board-style questions (pre 80% (60-100%) vs. post 100% (80-100%), W = 22, p = 0.17). No significant change in median test performance was seen in the traditional format (pre 90% (60-100%) vs. 90% (65-100%), W = 7, p = 0.68), while a non-significant increase was observed in the gamified format (70% (55-85%) vs. 100% (95-100%), W = 3, p = 0.5).

Combined Likert scale data assessing confidence in managing T2DM demonstrated a non-significant increase in median score (pre 3 (2.5-4) vs. post 4 (3.25-4), W = 21, p = 0.10). There were significant increases in Likert scale ratings of knowledge of the 2023 American Diabetes Association (ADA) Guidelines (pre 1 (1-2) vs. post 4 (3-4), W = 78, p < 0.01) and ratings of knowledge of the 2023 American Association of Clinical Endocrinology (AACE) Consensus Statement (pre 1 (1-2) vs. post 4 (3-4), W = 91, p < 0.01) [13,14].

Hypertension didactic

Around 16/108 (14.8%) total matched pairs in the gamified and traditional cohorts completed pre- and post-surveys. Combined data including both cohorts demonstrated a significant increase in median performance on board-style questions (pre 60% (40-80%) vs. post 80% (60-100%), W = 72, p = 0.02). When each cohort was assessed separately, non-significant increases in median test performance were seen in the traditional format (pre 70% (55-80%) vs. 100% (90-100%), W = 24, p = 0.06) and the gamified format (pre 60% (35-65%) vs. post 70% (55-80%), W = 14, p = 0.32).

Combined Likert scale data assessing confidence in managing hypertension demonstrated a significant increase in median score (pre 4 (3-4) vs. 4 (4-4.25), W = 55, p < 0.01). Significant increases were observed in Likert scale ratings of confidence in managing hypertension in chronic kidney disease and cardiovascular disease (pre 4 (2.75-4) vs. 4 (4-5), W = 45, p < 0.01), and ratings of knowledge of 2017 American College of Cardiology/American Heart Association (ACC/AHA) Guidelines (pre 3 (2-3.25) vs. post 4 (4-4.25), W = 91, p < 0.01) [15].

Hyperlipidemia didactic

Around 26/108 (24.0%) total matched pairs in the gamified and traditional cohorts completed pre- and post-surveys. Combined data including both cohorts demonstrated no significant change in median performance on board-style questions (pre 80% (60-80%) vs. 80% (60-100%), W = 50, p = 0.20). No significant changes were observed in median test performance in the traditional format (pre-80% (60-80%) vs. post-80% (65-100%), W = 15, p = 0.39), with a non-significant increase in the gamified format (pre 70% (60-85%) vs. 80% (60-85%), W = 11, p = 0.50).

Combined Likert scale data assessing confidence in managing hyperlipidemia demonstrated a significant increase in median score (pre 3 (3-4) vs. 4 (4-4), W = 154, p < 0.01). Significant increases were observed in Likert scale ratings of knowledge of the 2018 AHA/ACC Guideline [16] (pre 3 (2-3) vs. post 4 (3.25-4), W = 300, p < 0.01) and ratings of knowledge of the 2022 ACC Expert Consensus Decision Pathway (pre 2 (1-3) vs. post 4 (3-4), W = 276, p < 0.01) [16,17].

Metabolic-associated fatty liver dysfunction didactic

Around 41/108 (37.9%) total matched pairs in the gamified and traditional cohorts completed pre- and post-surveys. Combined data including both cohorts demonstrated a non-significant improvement in median performance on board-style questions (pre 50% (33.3-66.6%) vs. post 66.6% (33.3-83.3%), W = 68, p = 0.42). No significant changes were observed in median test performance in the traditional format (pre 50% (33.3-66.6%) vs. post 50% (41.6-83.3%), W = 37, p = 0.25) and the gamified format (pre 66.6% (50-79%) vs. post 66.6% (37.4-83.3%), W = -3, p = 0.94).

Combined Likert scale data assessing comfort evaluating transaminitis demonstrated significant increases in median score (pre 3 (3-4) vs. 4 (4-4), W = 231, p < 0.01). Significant increases were observed in Likert scale ratings of knowledge of American Association for the Study of Liver Diseases (AASLD) Practice Guidance [18] (pre 3 (2-3) vs. post 4 (3-4), W = 584, p < 0.01).

Weight management didactic

Around 20/108 (18.5%) total matched pairs in the gamified and traditional cohorts completed pre- and post-surveys. Combined data including both cohorts demonstrated no significant increase in median performance on board-style questions (pre 60% (60-60%) vs. post 60% (60-80%), W = 60, p = 0.13). There was a non-significant increase in median test performance in the traditional format (pre 60% (60-60%) vs. 80% (60-85%), W = 17, p = 0.12), and no change in the gamified format (pre 60% (55-65%) vs. post 60% (60-80%), W = 9, p = 0.79).

Combined Likert scale data assessing confidence in managing obesity demonstrated significant improvements in score (pre 3 (2-3) vs. post 4 (3-4), W = 55, p < 0.01). Increases were also seen in ratings of comfort in discussing weight loss medications (pre 3 (2-3) vs post 4 (3.75-4), W = 105, p < 0.01) and ratings of comfort in prescribing weight loss medications (pre 2.5 (2-3) vs. post 4 (3-4), W = 136, p < 0.01).

Analysis of Likert scale ratings across all five didactics within separate gamified and traditional cohorts demonstrated, in both cohorts, significant or near-significant increases from pre-test to post-test in ratings of confidence and knowledge of guidelines. Likert scale results are summarized in Table 3. Board-style question performance is summarized in Table 4.

**Table 3:** Likert scale assessments of confidence, comfort, and knowledge (combined data from both traditional and gamified cohorts)

Likert Scale Question	Matched Pairs (n)	Pre-test Likert Scale Score, From 1–5 (Median, (Range))	Post-test Likert Scale Score, From 1-5 (Median, (Range))	Wilcoxon Signed Rank Test (W)	Significance (p)
How confident are you in managing patients with type 2 diabetes mellitus?	14	3 (2.5-4)	4 (3.25-4)	W = 21	p = 0.10
How knowledgeable are you about the 2023 American Diabetes Association standards of care in diabetes?	14	1 (1-2)	4 (3-4)	W = 78	p < 0.01
How knowledgeable are you on the 2023 American College of Endocrinology and the American Association of Clinical Endocrinologists' comprehensive type 2 diabetes management algorithm?	14	1 (1-2)	4 (3-4)	W = 91	p < 0.01
How confident are you in managing patients with hypertension?	16	4 (3-4)	4 (4-4.25)	W = 55	p < 0.01
How confident are you in managing patients with hypertension and chronic kidney disease, or cardiovascular disease?	16	4 (2.75-4)	4 (4-5)	W = 45	p < 0.01
How knowledgeable are you about the 2017 American College of Cardiology/American Heart Association Guideline for the prevention, detection, evaluation, and management of high blood pressure in adults?	16	3 (2-3.25)	4 (4-4.25)	W = 91	p < 0.01
How confident are you in managing patients with dyslipidemia and cardiovascular disease or significant risk factors?	26	3 (3-4)	4 (4-4)	W = 154	p < 0.01
How knowledgeable are you about the 2018 American College of Cardiology guideline on the management of blood cholesterol?	26	3 (2-3)	4 (3.25-4)	W = 300	p < 0.01
How knowledgeable are you on the 2022 American College of Cardiology expert consensus decision pathway on the role of non-statin therapies in the prevention of atherosclerotic cardiovascular disease?	25	2 (1-3)	4 (3-4)	W = 276	p < 0.01
How comfortable are you working up patients with transaminitis (elevated liver function test (LFTs))?	41	3 (3-4)	4 (4-4)	W = 231	p < 0.01
How confident are you in referencing guidelines on managing cardiometabolic health and liver disease from professional societies such as the American Gastroenterological Association (AGA), the American Association of Clinical Endocrinology (AACE), or the American Association for the Study of Liver Diseases (AASLD)?	41	3 (2-3)	4 (3-4)	W = 584	p < 0.01
How confident are you in managing patients with obesity?	20	3 (2-3)	4 (3-4)	W = 55	p < 0.01
How comfortable are you discussing medications for weight loss?	19	3 (2-3)	4 (3.75-4)	W = 105	p < 0.01
How comfortable are you prescribing medications for weight loss?	20	2.5 (2-3)	4 (3-4)	W = 136	p < 0.01

**Table 4:** Knowledge assessment (performance on a set of standardized board-style internal medicine questions) MAFLD: metabolic-associated fatty liver dysfunction

Didactic Format	Matched Pairs (n)	Pre-Test Performance (Median, (Range))	Post-Test Performance (Median, (Range))	Wilcoxon Signed Rank Test (W)	Significance (p)
Type 2 diabetes mellitus combined data	14	80% (60-100%)	100% (80-100%)	W = 22	p = 0.17
Type 2 diabetes mellitus traditional format	10	90% (60-100%)	90% (65-100%)	W = 7	p = 0.68
Type 2 diabetes mellitus gamified format	4	70% (55-85%)	100% (95-100%)	W = 3	p = 0.5
Hypertension combined data	16	60% (40-80%)	80% (60-100%)	W = 72	p = 0.02
Hypertension traditional format	8	70% (55-80%)	100% (90-100%)	W = 24	p = 0.06
Hypertension gamified format	8	60% (35-65%)	70% (55-80%)	W = 14	p = 0.32
Hyperlipidemia combined data	26	80% (60-80%)	80% (60-100%)	W = 50	p = 0.20
Hyperlipidemia traditional format	14	80% (60-80%)	80% (65-100%)	W = 15	p = 0.39
Hyperlipidemia gamified format	12	70% (60-85%)	80% (60-85%)	W = 11	p = 0.50
MAFLD combined data	41	50% (33.3-66.6%)	66.6% (33.3-83.3%)	W = 68	p = 0.42
MAFLD traditional format	19	50% (33.3-66.6%)	50% (41.6-83.3%)	W = 37	p = 0.25
MAFLD gamified format	22	66.6% (50-79%)	66.6% (37.4-83.3%)	W = -3	p = 0.94
Weight loss combined data	20	60% (60-60%)	60% (60-80%)	W = 60	p = 0.13
Weight loss traditional format	8	60% (60-60%)	80% (60-85%)	W = 17	p = 0.12
Weight loss gamified format	12	60% (55-65%)	60% (60-80%)	W = 9	p = 0.79

Comparison of the change in pre-test and post-test knowledge

Analysis of differences in pre-post knowledge and confidence between cohorts was performed by comparing independent groups’ delta differences in median score on board-style questions and Likert scale ratings of confidence. Across all five didactics, the independent groups’ delta differences in test performance and Likert scale ratings of confidence were not significantly different between cohorts, suggesting that both gamified and traditional formats were equally efficacious in promoting post-curricular improvements in confidence and knowledge across all didactics.

End-of-year engagement survey

Of 18/108 (16.6%) residents who completed the survey, 11/18 (61.1%) received the gamified format and 7/18 (38.8%) received the traditional format. In the gamified cohort, 10/11 (90.9%) residents “strongly agreed” that the gamified curriculum as a whole was engaging, and 9/11 (81.8%) preferred to continue the format. In the traditional cohort, 1/7 (14.2%) residents “strongly agreed” that the traditional curriculum as a whole was engaging, and 4/7 (57.1%) preferred to continue the format. When reviewing the engagement data by individual didactic within the gamified cohort, 9/11 (81.8%) residents “strongly agreed” that each individual didactic was engaging. Within the traditional cohort, 1/7 (14.2%) residents “strongly agreed” that didactics on hyperlipidemia, hypertension, T2DM, and weight management were engaging, and 0/7 (0%) “strongly agreed” that the MAFLD didactic was engaging.

Residents generally “strongly agreed” that the didactics motivated them to adhere to guidelines. Within the gamified cohort, 9/11 (81.8%) “strongly agreed” that didactics on hyperlipidemia, hypertension, and T2DM motivated them, and 7/11 (63.6%) “strongly agreed” for weight management and MAFLD. Within the traditional group, 4/7 (57.1%) “strongly agreed” that the didactic on hyperlipidemia motivated them, 3/7 (42.8%) “strongly agreed” for hypertension and T2DM, 2/7 (28.5%) “strongly agreed” for MAFLD, and 1/7 (14.2%) “strongly agreed” for weight management.

## Discussion

Our study compared a novel gamified format using KAHOOT!® to a traditional slide-based format in a virtual cardiometabolic health curriculum, reviewing five key topics: T2DM, hypertension, hyperlipidemia, MAFLD, and weight management. We collected measures of learner engagement and self-reported Likert scale ratings of confidence in management and knowledge of guidelines. We also assessed knowledge by measuring performance on board-style questions. In designing our curriculum, we aimed to leverage multiple elements of gamification to improve the “fun” of learning guidelines with the ultimate goal of improving clinical care and resident adherence to professional association guidelines. By utilizing KAHOOT!®, multiple key elements of gamification were incorporated into the curriculum, including technology integration, a leaderboard, and point-based reward system, peer competition, and immediate feedback [19].

Residents demonstrated significant increases in post-curricular Likert scale ratings of confidence and knowledge in both the gamified and traditional cohorts, as well as in combined data including both cohorts. This demonstrates that, regardless of format, implementation of the curriculum increased resident confidence and knowledge in the management of cardiometabolic conditions and pertinent professional association guidelines. Contrary to what has been reported in the educational literature supporting greater learner confidence and motivation with a gamified format, we found no significant difference between a gamified and traditional format in improving ratings of confidence and knowledge [7].

Across all five didactics, there was no statistically significant increase in pre- to post-curricular test performance on board-style questions in either the combined cohort data or separate gamified and traditional cohort data. There was, however, a general trend to a higher median post-curricular test performance in both gamified and traditional cohorts. There was also no significant difference between cohorts in the change in test performance pre- to post-curriculum. As both gamified and traditional cohorts utilized case-based review, the benefit of both formats on educational outcomes and the lack of difference in test performance between a gamified and traditional format may simply support a general benefit of case-based review on learning outcomes, which has been observed previously [20]. A “ceiling effect,” where high baseline knowledge of board-style questions led to minimal pre-post change in performance, likely did not contribute to an observed lack of improvement, as pre-test performance ranged from 50-60% and post-test performance ranged from 70-80% across both formats and all five didactics. It is interesting to note that although residents reported significantly higher post-curricular ratings of confidence, this did not translate to higher test performance, which suggests greater subjective comfort that does not translate to improved post-curricular test scores.

What remains notable, however, is that a greater majority of residents found the gamified curriculum as a whole and its individual didactics engaging. Residents felt more motivated, with a gamified curriculum, in adhering to guidelines and generally preferred to continue a gamified format into the next academic year. This is consistent with a prior study by Gue et al. reporting greater ratings of motivation and engagement with a gamified system assigning points to in-person educational tasks throughout the academic year compared to a traditional format without a point-based system [21].

Limitations

The study was primarily limited by a single-center design and low response rate, which limits statistical power. However, our response rate was satisfactory in the context of prior literature in medical education, with response rates ranging from 20-30% [22]. Although curriculum attendance was mandatory, completing surveys was strictly voluntary. The decision to conduct voluntary surveys and include only residents who had completed both the pre- and post-surveys for matched analysis was a primary contributor to the low response rate. Response rate was highly variable between didactics, with the MAFLD didactic reporting the highest response rate (37.9%), possibly due to increased resident interest. Future avenues to improve response rate include specific incentives as well as more frequent reminders to complete surveys.

Data was not collected from non-respondents, likely due to the anonymous nature of surveys, which limited a comparison between respondents and non-respondents. It is possible that respondents could have been more engaged and motivated to learn compared to non-respondents. Our inclusion of Likert scale measures is limited by central tendency and acquiescence bias, and the interpretation of each scale may differ between residents. While Likert scales were utilized due to ease of data collection, a formal, validated measure could be utilized in the future. Additionally, surveys utilized a set of only five board-style questions, which limited granularity in measuring knowledge. Lengthier assessments with a greater number of questions would improve granularity in measuring knowledge. Additionally, a follow-up assessment would be useful in measuring long-term knowledge retention. Utilizing assessments with more questions, a larger sample size, and long-term follow-up would have increased statistical power to detect knowledge differences.

It remains unclear as to why increased engagement in the gamified format did not lead to greater improvement in test performance compared to a traditional format, which is to be expected based on prior studies [23]. We suspect that our limited response rate and granularity of assessments, along with a lack of long-term follow-up, limited our ability to assess the relationship between engagement and test performance. Additionally, it is possible that a gamified format simply improved the ease and enjoyment of learning rather than improving knowledge acquisition. A greater benefit may be seen from a more longitudinal curriculum with multiple follow-up review sessions, which would be an interesting future avenue of study. Faculty satisfaction was not measured within our study, but also warrants further investigation as the benefits of gamification on faculty satisfaction in teaching have been reported in the literature [3,5].

## Conclusions

A gamified format was similar to a traditional format in improving self-perceived and objective knowledge of cardiometabolic health. While there was no advantage of a gamified format in improving test performance, residents undergoing a gamified format felt more engaged and motivated to utilize practice guidelines in clinical practice. This study is notable as it is one of the few that evaluates the benefits of an SRS as a gamification system on the acquisition of medical knowledge. Additionally, to our knowledge, it is one of the first that evaluates a cohesive virtual gamified curriculum comprising multiple topics within one subject area. As a result, our study provides novel data assessing a virtual didactic format utilizing SRS that supplements existing educational literature assessing in-person didactic formats utilizing alternative gamification systems such as board games, roleplay, or assigning points to educational tasks.

## Figures and Tables

**Figure 1:**
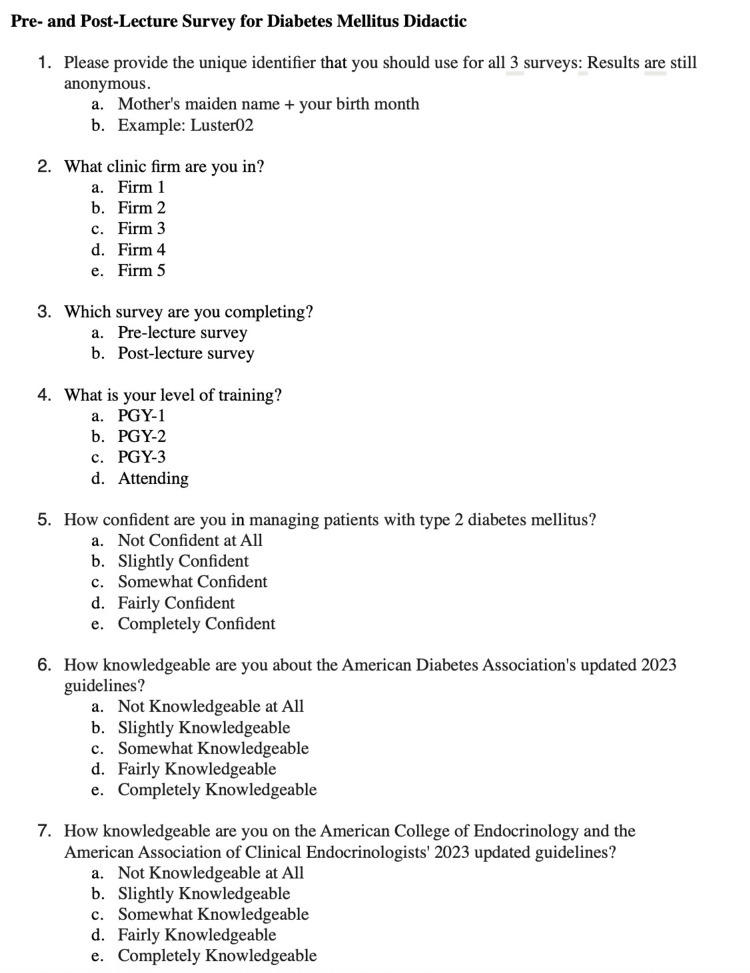
Survey (part 1)

**Figure 2:**
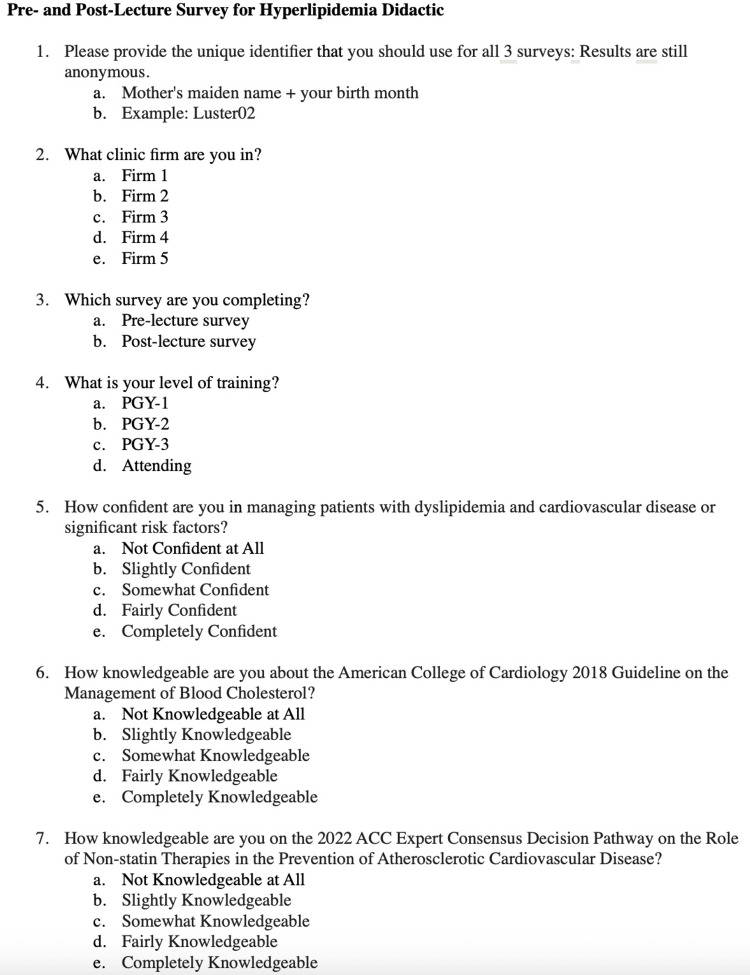
Survey (part 2) ACC: ACC: American College of Cardiology

**Figure 3:**
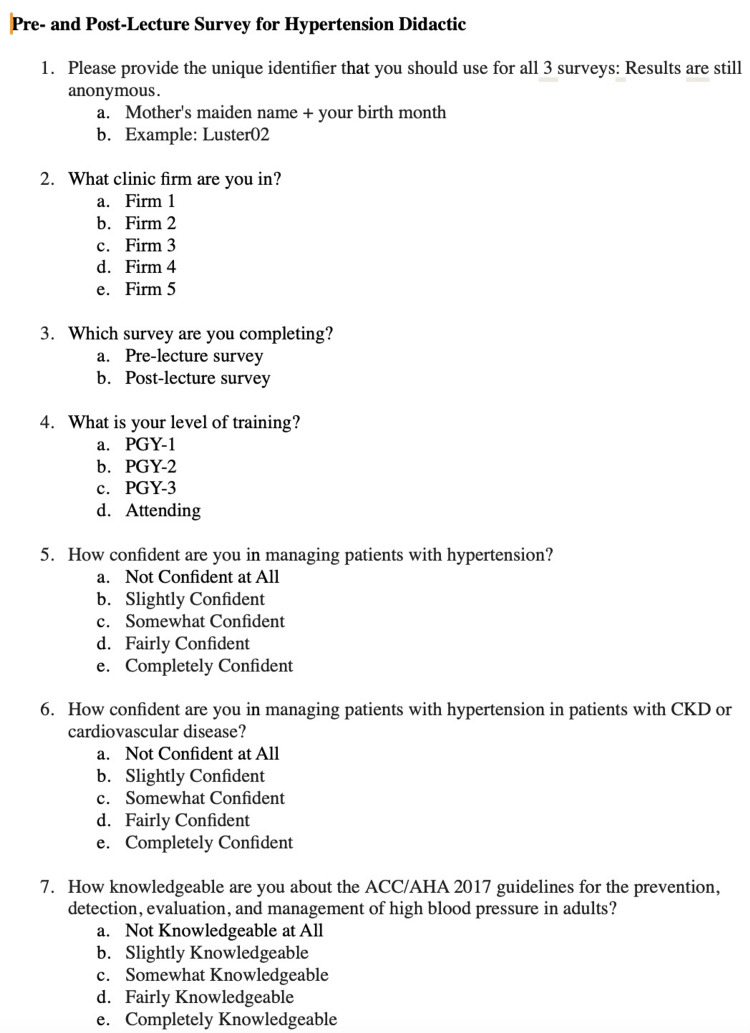
Survey (part 3) CKD: chronic kidney disease; AHA: American Heart Association; ACC: American College of Cardiology

**Figure 4:**
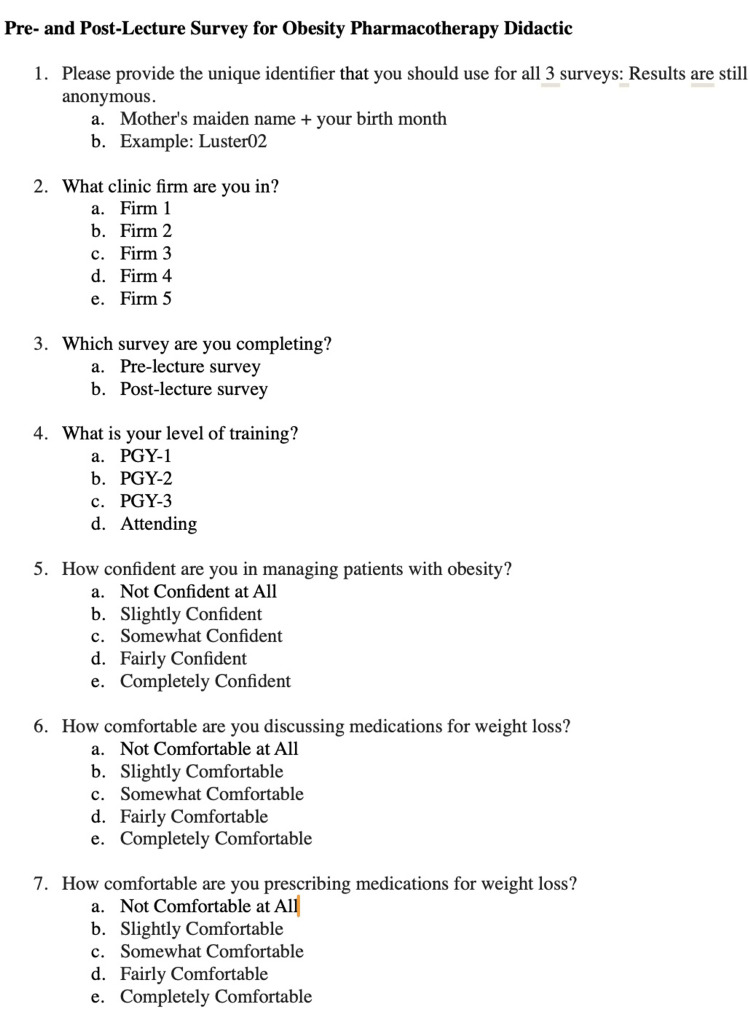
Survey (part 4)

**Figure 5:**
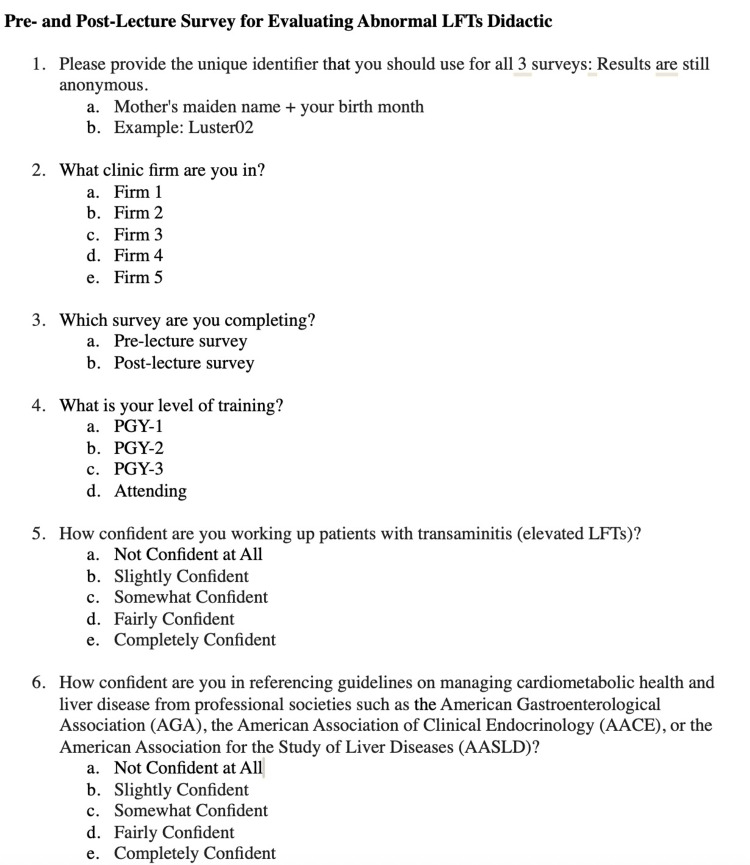
Survey (part 5) LFT: liver function test

**Figure 6:**
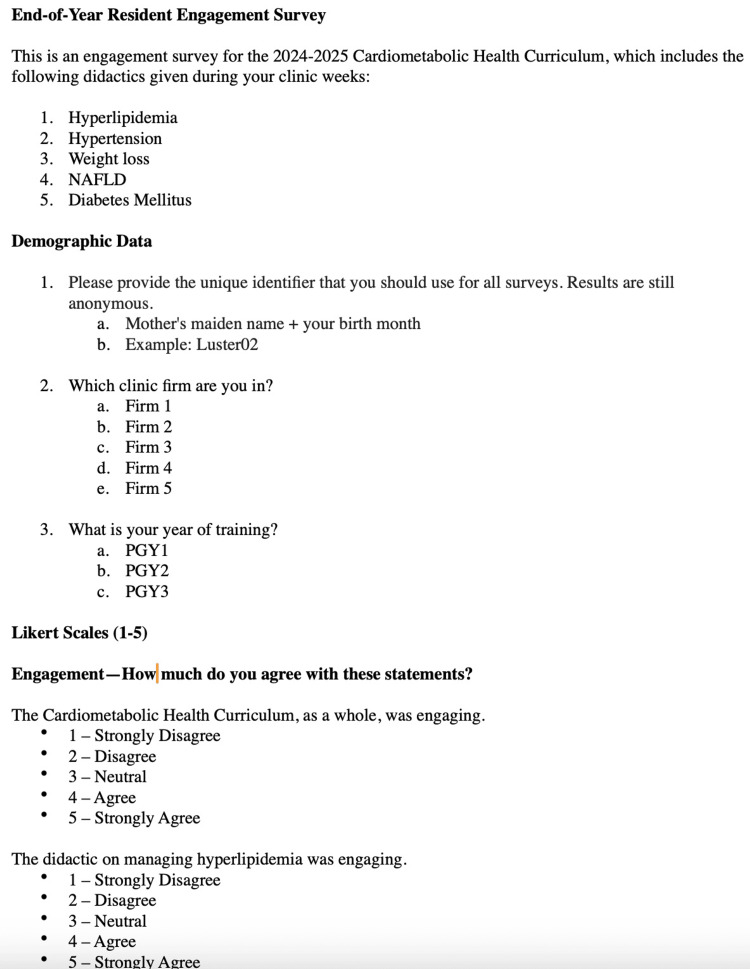
Survey (part 6)

**Figure 7:**
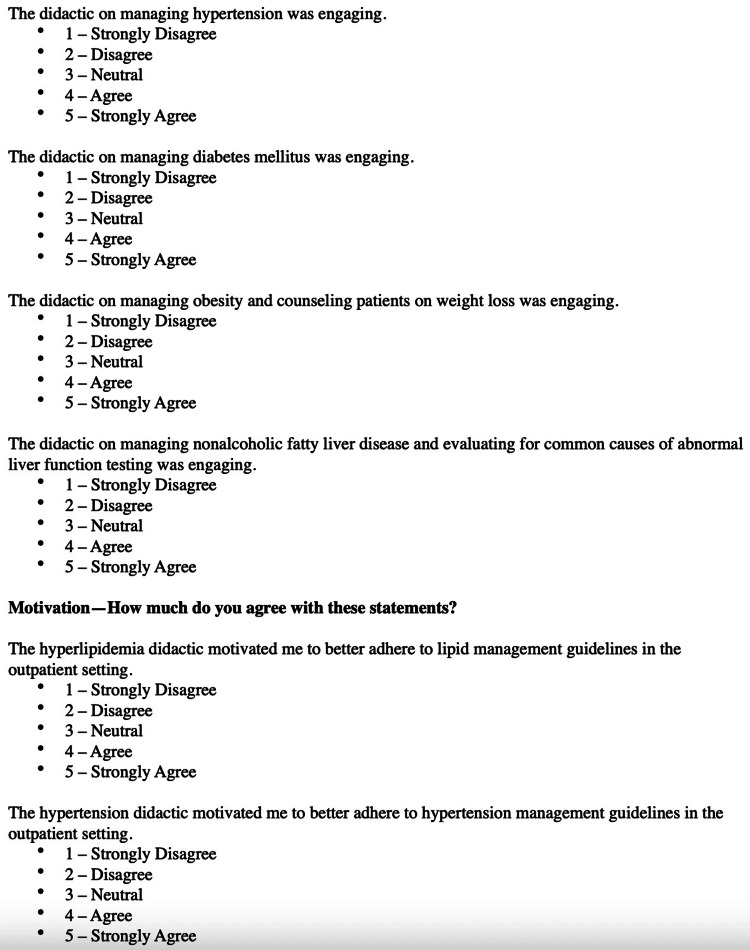
Survey (part 7)

**Figure 8:**
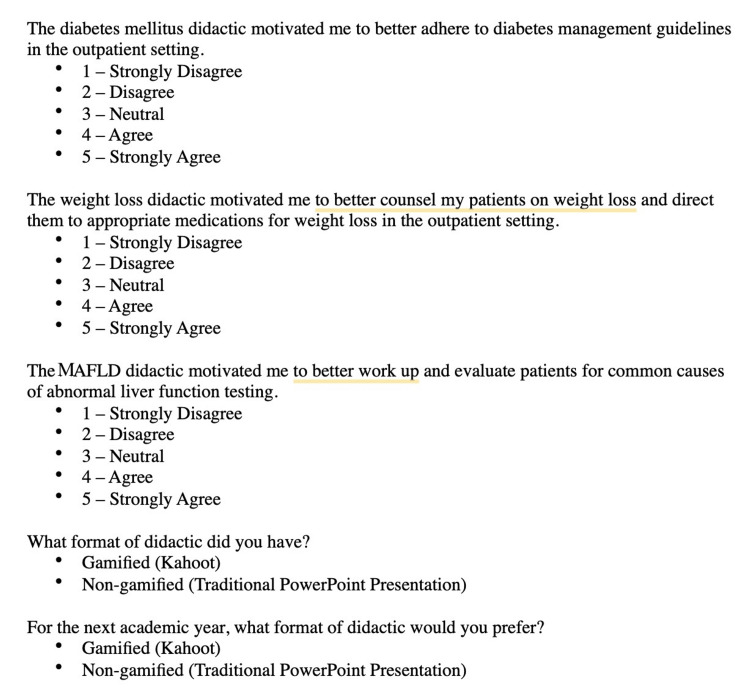
Survey (part 8) MAFLD: metabolic-associated fatty liver dysfunction

## Appendices

Appendix 1
